# Dengue fever in a liver-transplanted patient: a case report

**DOI:** 10.1186/1752-1947-8-378

**Published:** 2014-11-21

**Authors:** Ranga Migara Weerakkody, Dhammika Randula Palangasinghe, Kaluthanthri Patabandi Chamila Dalpatadu, Jeewan Pradeep Rankothkumbura, Mohammed Rezni Nizam Cassim, Panduka Karunanayake

**Affiliations:** 1 University Medical Unit, National Hospital of Sri Lanka, Regent Street, Colombo 9 CO00900, Sri Lanka; 2 Department of Surgery, Faculty of Medicine, University of Colombo, Kynsey Road, Colombo 8 CO00800, Sri Lanka; 3 Department of Clinical Medicine, Faculty of Medicine, University of Colombo, Kynsey Road, Colombo 8 CO00800, Sri Lanka

**Keywords:** Dengue, Liver transplant, NS1 antigen, Tacrolimus toxicity

## Abstract

**Introduction:**

Dengue fever is one of the commonest mosquito-borne diseases in the tropics, and Sri Lanka is no exception. Despite its commonness, dengue fever has rarely been described among patients who have undergone transplantation. We report the case of a patient with dengue fever after liver transplantation, which, to the best of our knowledge, is the first such reported case outside Brazil.

**Case presentation:**

Our patient was a 46-year-old Sri Lakan man who presented to our institution two years after undergoing an ABO-compatible cadaveric liver transplant. At presentation, he had typical symptoms of dengue fever. He was taking prednisolone 5mg daily and tacrolimus 3mg twice daily as immunosuppression. Initial investigations showed thrombocytopenia and neutropenia that reached a nadir by day 7 of his illness. He had elevated liver enzymes as well. The diagnosis was confirmed on the basis of NS1 antigen detection by enzyme-linked immunosorbent assay. His blood cultures and polymerase chain reaction tests for cytomegalovirus were negative. He made an uneventful recovery and was discharged by day 9 of his illness. However, normalization of liver function took nearly two weeks. In three previously reported Brazilian cases of dengue after liver transplantation, the patients presented with dengue shock syndrome, in contrast to the relatively milder presentation of our patient. Because of the lack of case reports in the literature, it is difficult to ascertain the risk factors for severe dengue infection in transplants, but dengue fever reported in renal transplants sheds some light on them. High-dose steroids increase the risk of thrombocytopenia, whereas tacrolimus has been reported to prolong the duration of symptoms. Otherwise, dengue fever is a relatively mild illness in patients who have undergone renal transplantation, and renal allograft survival has been reported to be 86% following dengue fever.

**Conclusion:**

Dengue is a rarely reported infection in patients who have undergone transplantation. A high degree of suspicion is required for diagnosis. Dengue NS1 antigen detection is a useful addition to the already existing methods of diagnosis. Steroids and tacrolimus have effects on the morbidity of the disease. Graft outcomes following the infection has been excellent in all reported cases.

## Introduction

Dengue fever (DF) is one of the commonest mosquito-borne diseases in the tropics and has become hyperendemic in Sri Lanka. Although thousands of cases are reported yearly in Sri Lanka, dengue has been a rarely reported disease in patients who have undergone transplantation [1]. We describe the case of a patient with DF after a liver transplant (LT). To the best of our knowledge, this is the first such reported case outside Brazil.

## Case presentation

A 46-year-old Sri Lankan man presented to our institution with high-grade fever associated with severe retro-ocular pain, photophobia, bone pain and severe body aches of three days’ duration. He was a recipient of an ABO-compatible cadaveric LT 26 months previously for treatment of end-stage cryptogenic cirrhosis. The immediate post-transplant period had been complicated by acute rejection, but he made a full recovery with an allograft. He was maintained on prednisolone 5mg daily and tacrolimus 3mg twice daily. He had developed diabetes after the third month post-LT. His two sons had had a febrile illness, diagnosed as DF, one week before coming to our institution. He did not complain of respiratory, urinary or bowel symptoms or a rash. An examination did not reveal jaundice, rashes or neck stiffness, but demonstrated a fine tremor in outstretched hands. His right frontal sinus was tender on palpation.

**Figure 1:**
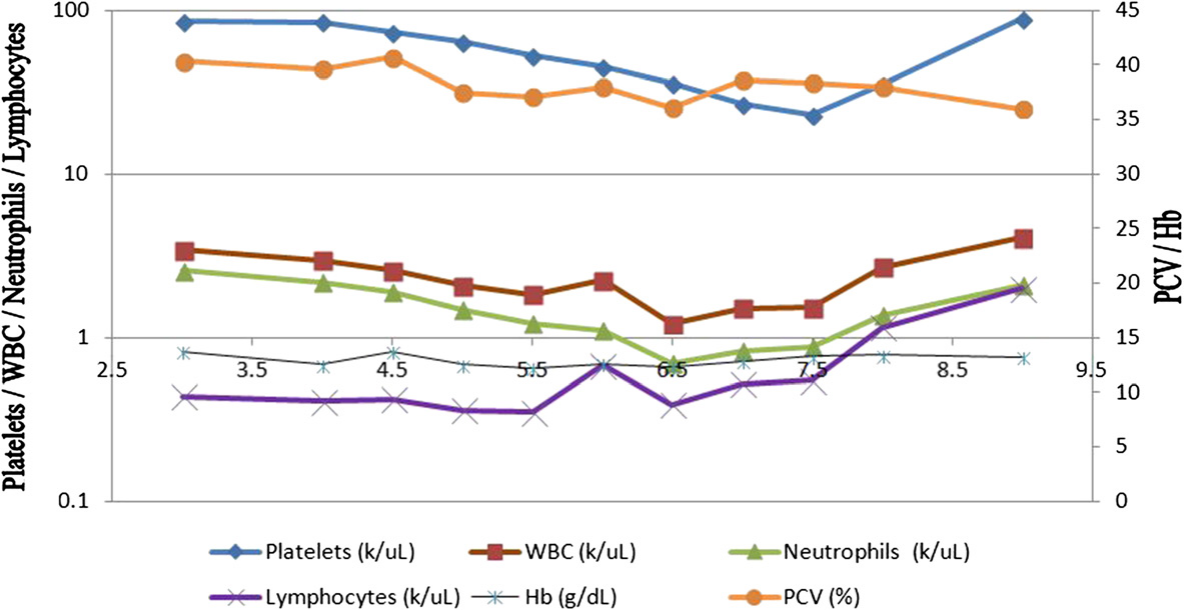
**Graphical representation of changing hematological and biochemical parameters.** Hb, Hemoglobin; PCV, Packed cell volume; WBC, White blood cell.

His initial full blood count revealed thrombocytopenia (90,000/μL), and, with a suggestive clinical picture, a provisional diagnosis of DF was made. His skull X-ray demonstrated a fluid level in the right frontal sinus confirming acute sinusitis, which was treated with intravenous ceftriaxone 1g twice daily. Over the next few days, his white cell count and platelet count gradually dropped, reaching a nadir by day 4 of the illness. Dengue NS1 antigen was detected in his day 3 serum sample, confirming the diagnosis of DF. His liver enzymes rose sharply until day 7 before starting to recover. His bilirubin levels and coagulation profile were normal. He entered the leakage (critical) phase on day 6 of his illness, and he recovered by day 8 without any complications with standard supportive DF management. DNA detection of cytomegalovirus was negative, and his blood cultures were sterile. However, liver enzyme normalization took more than two weeks. Throughout his hospital stay, his serum creatinine was around 140μmol/L and, together with fine tremors, was suggestive of tacrolimus toxicity (T_0_ = 13.4ng/dL). Tacrolimus was reduced to 4mg daily as a result. His creatinine levels dropped to 104μmol/L by the time of discharge. The day 7 and day 14 dengue immunoglobulin M (IgM) titers showed an eightfold increase. Figure 1 shows the changes in parameters over time.

## Discussion

DF is the most widespread mosquito-borne disease in Sri Lanka. Despite the high prevalence, it is a rarely reported disease in patients who have received solid-organ transplants. Patients who have undergone transplantation may have diminished T-cell responses and may be at low risk for contracting DF, which perhaps accounts for the lack of clinical reports describing such patients [2]. In addition, there are relatively few patients living with allografts in Sri Lanka. Our literature search showed only three cases, all of which were in Brazil [3,4] and were diagnosed as dengue shock syndrome (DSS). One of these patients had jaundice, a lowest platelet count of 30,000/μL and extremely high liver enzyme levels in the range of 10^4^U/L. The laboratory examination findings for the other two previously reported patients are not available. Our patient had lower platelet counts than these patients, but his liver enzymes were much lower and he never had jaundice. One of the previously reported patients developed DSS three weeks following the transplant [4], and the other two contracted it in the ninth month post-transplantation [3]. Contrary to the pattern in the previously reported cases, our patient developed the illness two years after transplantation. The clinical picture was that of DF, not DSS as in the previously reported cases. Our patient had a fever pattern that is different from that seen in normal DF, by virtue of a prolonged febrile phase, which ran up to one week. Superadded sinusitis may have had an effect on the length of the febrile phase. Photophobia is an uncommon symptom of DF, but dengue meningoencephalitis can present with that symptom. Sinus tenderness and an abnormal X-ray view of the sinuses makes diagnosis of acute frontal sinusitis more likely. A lumbar puncture and CSF analysis would have been extremely useful in this situation, but the rapid resolution of symptoms with antibiotics and in the face of thrombocytopenia, lumbar puncture was not tried.

Although data on LT are limited, data from a large case series on patients who received renal transplants (RTs) have been reported [5]. Nasim and colleagues reported that the mean duration of high-grade fever was 3.4 ± 1.5 days and that of low-grade fever was 6.0 ± 3.5 days. Our patient went through a febrile phase that was similar in length, but his fever was high-grade. In the patients described by Nasim and colleagues, severe primary disease was seen more often in patients receiving high doses of steroids (>7.5mg/d) compared to low-dose regimens (≤7.5mg/d). Thrombocytopenia was most severe in patients receiving steroids, azathioprine (AZA) and cyclosporin A (CyA) concomitantly. Mycophenolate mofetil (MMF) or AZA had no effect on the severity of thrombocytopenia, whereas tacrolimus prolonged the duration of thrombocytopenia compared to MMF or AZA alone or to either one in combination with CyA [5]. The deaths that occurred were not related to DF, and graft survival was excellent (86%) following the infection.

Diagnosing DF is challenging in patients who have undergone transplantation, owing to immunosuppression, which may suppress some of the cardinal features of DF. The diagnosis may be delayed if fever and thrombocytopenia are initially attributed to co-morbidities such as cytomegalovirus sepsis or because of anti-mitotic agents or hepatitis C virus [5]. It is prudent to believe that most of the infections in patients with immunosuppression may be subclinical and indistinguishable from a non-specific viral fever and, as a result, may never be reported. The actual infection rates could be much higher than reported. Additionally, the diagnosis in the previously reported cases, as well as in series where RT was involved, were arrived at using antibody (IgM) detection. In comparison, we used both NS1 antigen detection and IgM titer. Dengue NS1 antigen detection has better sensitivity and specificity than other methods of detection of dengue virus [6] and provide confirmation of the diagnosis as early as day 3 of illness. We believe that use of antigen detection as a screening test will help to diagnose more cases of DF in patients who have received transplants. In almost all patients with DF, the infection is vector-borne. Interestingly, hematogenic spread of dengue has also been reported in patients who have undergone transplantation [7], from the donor via the allograft, which is the only record of mortality.

Data on sequelae following DF in LT patients are scarce because of the extremely limited number of cases reported in the literature to date. Data from case series involving RT patients show no increase in graft failures or episodes of acute rejection [8]. Although it included no patients with DF who had received LTs, a Taiwan series showed an increased incidence of biliary atresia following a DF epidemic in 2002 [9].

## Conclusions

DF is a rarely reported infection in transplanted patients and requires a high degree of suspicion for proper diagnosis. Dengue NS1 antigen detection is a useful addition to the already existing methods of diagnosis. Steroids and tacrolimus increase the morbidity of the disease. The graft outcome following the infection in our patient has been excellent, as in previously reported cases.

## Consent

Written informed consent was obtained from the patient for publication of this case report and any accompanying images. A copy of the written consent is available for review by the Editor-in-Chief of this journal.

## Abbreviations

AZA: Azathioprine; CyA: Cyclosporin A; DF: Dengue fever; DSS: Dengue shock syndrome; LT: Liver transplant; MMF: Mycophenolate mofetil; NS1: Nonspecific antigen 1; RT: Renal transplant.

## Competing interests

The authors declare that they have no competing interests.

## Authors’ contributions

The patient was under the primary care of MRNC for liver transplantation. PK was the primary care physician under whom the patient was admitted to our institution and was the supervisor of DRP, KPCD and JPR. DRP, KPCD and JPR were the team who took care of the patient’s primary illness. RMW designed the manuscript, did the literature survey and helped with immunosuppression modifications. DRP and JPR wrote the manuscript, and RMW and KPCD critically analyzed it. All authors read and approved the final manuscript.
